# A Rate-Distortion-Based Merging Algorithm for Compressed Image Segmentation

**DOI:** 10.1155/2012/648320

**Published:** 2012-10-15

**Authors:** Ying-Shen Juang, Hsi-Chin Hsin, Tze-Yun Sung, Yaw-Shih Shieh, Carlo Cattani

**Affiliations:** ^1^Department of Business Administration, Chung Hua University, Hsinchu City 30012, Taiwan; ^2^Department of Computer Science and Information Engineering, National United University, Miaoli 36003, Taiwan; ^3^Department of Electronics Engineering, Chung Hua University, Hsinchu City 30012, Taiwan; ^4^Department of Mathematics, University of Salerno, Via Ponte Don Melillo, 84084 Fisciano, Italy

## Abstract

Original images are often compressed for the communication applications. In order to avoid the burden of decompressing computations, it is thus desirable to segment images in the compressed domain directly. This paper presents a simple rate-distortion-based scheme to segment images in the JPEG2000 domain. It is based on a binary arithmetic code table used in the JPEG2000 standard, which is available at both encoder and decoder; thus, there is no need to transmit the segmentation result. Experimental results on the Berkeley image database show that the proposed algorithm is preferable in terms of the running time and the quantitative measures: probabilistic Rand index (PRI) and boundary displacement error (BDE).

## 1. Introduction

Data segmentation is important in many applications [1–6]. Early research work on image segmentation is mainly at a single scale, especially for medical images [7–9]. In the human visual system (HVS), the perceived image is decomposed into a set of band-pass subimages by means of filtering with simple visual cortical cells, which can be well modeled by Gabor filters with suitable spatial frequencies and orientations [10]. Other state-of-the-art multiscale techniques are based on wavelet transform (WT), which provides an efficient multiresolution representation in accord with the property of HVS [11]. Specifically, the higher-detail information of an image is projected onto a shorter basis function with higher spatial resolution. Various WT-based features and algorithms were proposed in the literature for image segmentation at multiple scales [12–14].

For the communication applications, original images are compressed in order to make good use of memory space and channel bandwidth. Thus, it is desirable to segment a compressed image directly. The Joint Photographic Expert Group (JPEG) standard adopts discrete cosine transform for subband image coding. In order to improve the compression performance of JPEG with more coding advantages, for example, embedded coding and progressive transmission, the JPEG2000 standard adopts WT as the underlying transform algorithm. Specifically, embedding coding is to code an image into a single code stream, from which the decoded image at any bit rate can be obtained. The embedded code stream of an image is organized in decreasing order of significance for progressive transmission over band-limited channels. This property is particularly desirable for the Internet streaming and database browsing applications [15–17]. Zargari proposed an efficient method for JPEG2000 image retrieval in the compressed domain [18]. Pi proposed a simple scheme to estimate the probability mass function (PMF) of wavelet subbands by counting the number of 1-bits and used the global PMF as features to retrieve similar images from a large database [19]. For image segmentation, however, the local PMF is needed. In [20], we proposed a simple method to compute the local PMF of wavelet coefficients based on the MQ table. It can be applied to a JPEG2000 code stream directly, and the local PMF can be used as features to segment a JPEG2000 image in the compressed domain.

Motivated by the idea behind the postcompression rate distortion (PCRD) algorithm [15], we propose a simple algorithm called the rate-distortion-based merging (RDM) algorithm for JPEG2000 image segmentation. It can be applied to a JPEG2000 code stream instead of the decoded image. As a result, the burden of decoding computation can be saved. In addition, the RDM algorithm is based on the MQ table, which is available at both encoder and decoder; thus, no overhead transmission is added from a segmentation viewpoint. The remainder of the paper proceeds as follows. In Section 2, the JPEG2000 standard is reviewed briefly. In Section 3, the MQ-table-based rate distortion slope (MQRDS) is proposed to examine the significance of wavelet segments; based on which, the RDM algorithm is thus proposed to merge wavelet segments with similar characteristics. Experimental results on the Berkeley color image database are given in Section 4. Conclusions can be found in Section 5.

## 2. Review of the JPEG2000 Standard

The core module of the JPEG2000 standard is the embedded block coding with optimized truncation (EBCOT) algorithm [15], which adopts wavelet transform (WT) as the underlying method to decompose an image into multiresolution subbands. WT has many desirable properties, for example, the self-similarity of wavelet coefficients across subbands of the same orientation, the joint space-spatial frequency localization with orientation selectivity, and the energy clustering within each subband [11]. The fundamental idea behind EBCOT is to take advantage of the energy clustering property of wavelet coefficients. EBCOT is a two-tier algorithm; tier-1 consists of bit plane coding (BPC) followed by arithmetic coding (AC); tier-2 is primarily for optimal rate control. Three coding passes, namely, the significance propagation (SP) pass, the magnitude refinement (MR) pass, and the clean-up (CU) pass, are involved with four primitive coding operations, namely, the significance coding operation, the sign coding operation, the magnitude refinement coding operation, and the clean-up coding operation. For a wavelet coefficient that is currently insignificant, if any of the 8 neighboring coefficients are already significant, it is coded in the SP pass using the significance coding operation; otherwise, it is coded in the CU pass using the clean-up coding operation. If this coefficient becomes significant, its sign is then coded using the sign coding operation. The magnitude of the significant wavelet coefficients that have been found in the previous coding passes is updated using the magnitude refinement coding operation in the MR pass. The resulting code streams of coding passes can be compressed further by using a context-based arithmetic coder known as the MQ coder. JPEG2000 defines 18 context labels for the MQ coder and stores their respective probability models in the MQ table. Specifically, 10 context labels are used for the significance coding operation and the clean-up coding operation, 5 context labels are used for the sign coding operation, and 3 context labels are used for the magnitude refinement coding operation.

In JPEG2000, a large image can be partitioned into nonoverlapped subimages called tiles for computational simplicity. WT is then applied to the tiles of an image for subband decompositions; and each wavelet subband is further divided into small blocks called code blocks. The code blocks of an image are independently coded from the most significant bit plane (MSB) to the least significant bit plane (LSB). Based on the true rate-distortion slope (RDS) of code blocks, JPEG2000 concatenates the significant code streams with large RDS using the post compression rate distortion (PCRD) algorithm for optimal rate control. More specifically, let {*B*_*i*_} be a set of code blocks in the whole image. The code stream of *B*_*i*_ can be terminated at the end of a coding pass, say *n*_*i*_, with the bit rate denoted by *R*_*i*_^*n*_*i*_^; all the end points of coding passes are possible truncation points. The distortion incurred by discarding the coding passes after *n*_*i*_ is denoted by *D*_*i*_^*n*_*i*_^. PCRD selects the optimal truncation points to minimize the overall distortion: *D* = ∑_*i*_ *D*_*i*_^*n*_*i*_^ subject to the rate constraint: *R* = ∑_*i*_ *R*_*i*_^*n*_*i*_^ ≤ *R*_*c*_, where *R*_*c*_ is a given bitrate. It is noted that the coding passes with nonincreasing RDS are candidates for the optimal truncation points. Motivated by the idea of the above, a new technique is proposed to segment JPEG2000 images in the JPEG2000 domain; the detail is given in the following section.

## 3. Image Segmentation in the JPEG2000 Domain

This section presents a simple merging algorithm for JPEG2000 image segmentation. It merges wavelet segments with similar characteristics based on the change of the estimated RDS in the JPEG2000 domain. Thus, the proposed algorithm can be applied to a JPEG2000 code stream without decompressing complexity.

### 3.1. MQ Table-Based Probability Mass Function

In JPEG2000, the wavelet coefficients of an image are quantized with bit planes, and binary wavelet variables are almost independent across bit planes. The probability mass function (PMF) known as the wavelet histogram [19] can be approximated by

(1) $$\begin{array}{c} P\left(\left|c\right|=x\right)=\prod_{j=0}^{n-1}P_{j}\left(x_{j}\right),\\ x=\sum_{j=0}^{n-1}x_{j}\cdot 2^{j};\;x_{j}\in \left\{0,1\right\}, \end{array}$$

where *x* is the magnitude of a wavelet coefficient,  *c*, *P*_*j*_(∘) is the PMF of the binary wavelet variable, *x*_*j*_, on the *jth* bit plane, and *n* is the number of bit planes. For image segmentation, the local PFM is needed. We had proposed a simple method to estimate the local PMF based on the MQ table [20]. Specifically, the probability of 1-bit, *P*_*j*_(*x*_*j*_ = 1), is given by

(2) $$\begin{array}{c} P_{j}\left(x_{j}=1\right)=\{\begin{array}{cc} Qe_{-}\text{Value} & \text{if}\;\;\text{MPS}=0,\\ 1-Qe_{-}\text{Value} & \text{if}\;\;\text{MPS}=1, \end{array} \end{array}$$

where *Qe*_−_Value is the probability of the less probable symbol (LPS), which is stored in the MQ table and MPS denotes the more probable symbol. The set {*P*_*j*_(*x*_*j*_ = 1); *j* = 0,…, *n* − 1} obtained from the MQ table can be used to compute the local PMF. As the MQ table is also available at decoder, no overhead transmission is needed for the computation of PMF. In addition, JPEG2000 defines only 18 context labels to model the binary wavelet variables; thus, the computation of PMF is simple.

### 3.2. MQ Table-Based Rate Distortion Slope and Merging Algorithm

Motivated by the post compression rate distortion (PCRD) algorithm [15], we propose the MQ table-based rate distortion slope (MQRDS) for image segmentation in the JPEG2000 domain as follows:

(3) $$\begin{array}{c} S_{m}=\frac{E\left[D_{m}\right]}{E\left[L_{m}\right]}, \end{array}$$

where *D*_*m*_ is the distortion of wavelet segment: *m* defined as

(4) $$\begin{array}{c} D_{m}=\sum_{i=1}^{N_{m}}x_{m,i}^{2}, \end{array}$$

*x*_*m*,*i*_ is a wavelet coefficient at location: *i* in wavelet segment, *m* represented by

(5) $$\begin{array}{c} x_{m,i}=\sum_{j=0}^{n-1}x_{m,i,j}\cdot 2^{j};\;x_{m,i,j}\in \left\{0,1\right\}. \end{array}$$

The estimate of *D*_*m*_ can be computed by

(6) E[Dm]=∑i=1Nm ∑j=0n−1 ∑k=0n−1E[xm,i,j·xm,i,k]·2j+k≅∑i=1Nm ∑j=0n−1 ∑k=0n−1E[xm,i,j]·E[xm,i,k]·2j+k,

in which *E*[*x*_*m*,*i*,*j*_] can be obtained from the binary arithmetic code table known as the MQ table as follows:

(7) $$\begin{array}{c} E\left[x_{m,i,j}\right]=P_{m,i,j}\left(x_{m,i,j}=1\right). \end{array}$$

The estimate of code length *E*[*L*_*m*_] can be efficiently obtained by using [2]

(8) $$\begin{array}{c} E\left[L_{m}\right]=\left(D+N_{m}\right)\cdot E\left[R_{m}\right]-N_{m}\text{lo}{\text{g}}_{2}\left(\frac{N_{m}}{N}\right) \end{array}$$

(9) $$\begin{array}{c} E\left[R_{m}\right]=\sum_{j=0}^{n-1}H\left(x_{m,j}\right), \end{array}$$

(10) H(xm,j)=−Pm,j(xm,j=1)·log2(Pm,j(xm,j=1))−Pm,j(xm,j=0)·log2(Pm,j(xm,j=0)),

(11) $$\begin{array}{c} P_{m,j}\left(x_{m,j}\right)=\frac{1}{N_{m}}\sum_{i=1}^{N_{m}}\cdot P_{m,i,j}\left(x_{m,i,j}\right), \end{array}$$

where *j* denotes the bit plane index, *x*_*m*,*i*,*j*_ is the binary variable of *x*_*m*,*i*_ on bit plane *j*, which are independent across bit planes, *n* is the number of bit planes, *D* is the feature space dimension, *N*_*m*_ is the number of wavelet coefficients in segment *m*, *N* = ∑_*m*=1_^*K*^ *N*_*m*_ is the total number of wavelet coefficients, and *H*(∘) is an entropy operation. After merging two wavelet segments, say *m* and *n*, the change of MQRDS is given by

(12) ΔSmn=[Smn−((Nm/(Nm+Nn  ))Sm+(Nn/(Nm+Nn  ))Sn)]Smn,

where *S*_*m*_ and *S*_*n*_ are the MQRDS of wavelet segments, *m* and *n*, with sizes *N*_*m*_ and *N*_*n*_, respectively, and *S*_*mn*_ is the MQRDS of the merged wavelet segment. As one can see, the change of MQRDS is likely to be increased significantly for wavelet segments with similar characteristics. Thus, we propose a simple algorithm called the rate-distortion-based merging (RDM) algorithm for JPEG2000 image segmentation, which is presented in the steps below.

**The RDM Algorithm**

 
*Step *1. Given a JPEG2000 code stream, compute the MQ table-based local PMF of wavelet coefficients using (2).
 
*Step *2. As mentioned in [2], a set of oversegmented regions known as superpixels is in general needed for any merging algorithms; this low-level initial segmentation can be obtained by coarsely clustering the local PMF as features.
 
*Step *3. For all pairs of superpixels, compute their respective changes of MQRDS using (12), and merge the one with maximum change of MQRDS.
 
*Step *4. Continue the merging process in step 3 until the change of MQRDS is insignificant.

In order to reduce the computation time, the following equation can be used to approximate (6):

(13) E[Dm]≅Nm·[∑j=0n−1 ∑k=0n−1(1Nm∑i=1NmPm,i,j(xm,i,j=1))   ·(1Nm∑i=1NmPm,i,k(xm,i,k=1))·2j+k].

**Figure 1:**
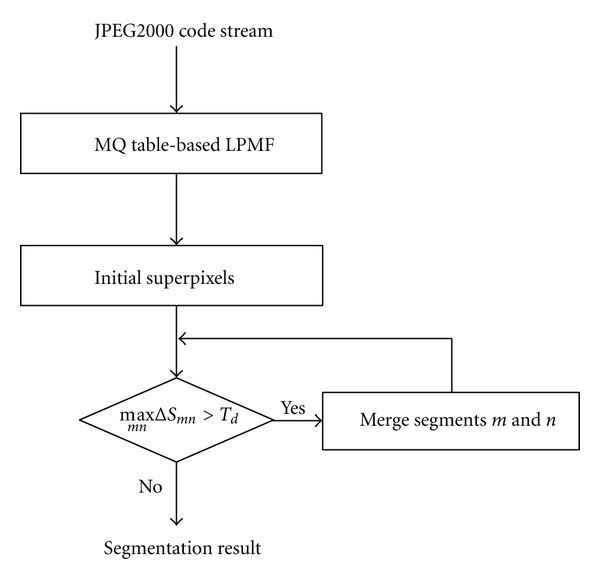
Flowchart of the RDM algorithm.

**Figure 2:**
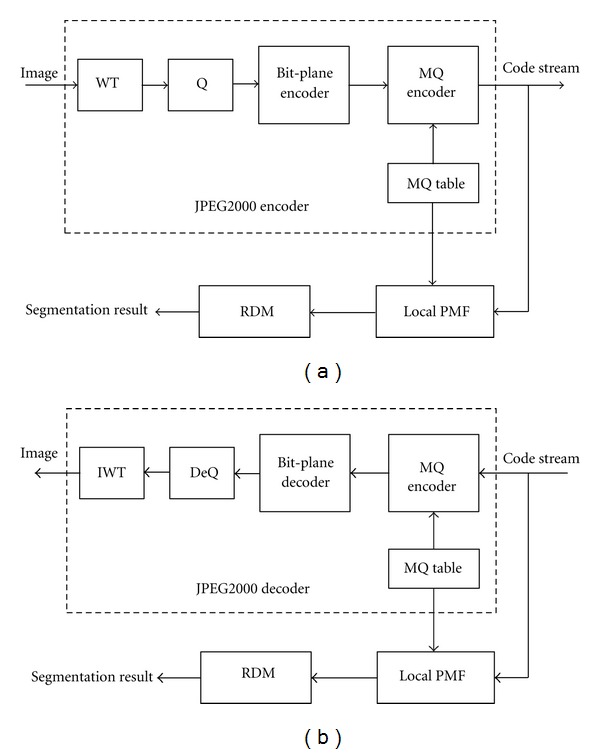
Image segmentation using RDM in the JPEG2000 domain; (a) encoder; (b) decoder.

Moreover, the cross terms of the previous equation are not significant and can be discarded for computational simplicity.  Figure 1 depicts flowchart of the RDM algorithm. It is noted that the MQ table defined in JPEG2000 is finite, thus (10) can be obtained by look-up table (LUT); this sure reduces the computation time further. As shown in Figure 2, RDM can be applied to a JPEG2000 code stream directly; this is one of the advantages of RDM.

## 4. Experimental Results

**Figure 3:**
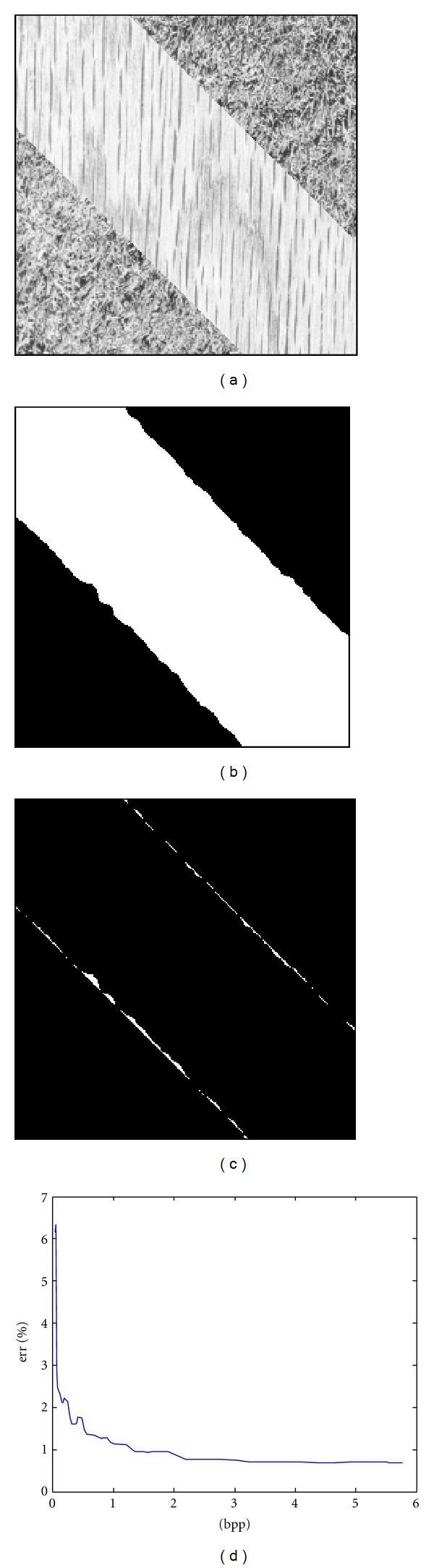
(a) Test image; (b) the segmentation result and (c) error image at 1 bpp; (d) error rates in percentage at various bpp rates.

In the first experiment, the potential of the MQ table-based local PMF (LPMF) is shown by segmenting images with Brodatz textures. As noted, the essential characteristics of textures are mainly contained in the middle-high-frequency wavelet subbands; thus, we applied a simple clustering algorithm known as K-means to the LPMF of wavelet coefficients to generate an initial segmentation. The number of superpixels was set to 30, which was then finely merged using the RDM algorithms.  Figure 3(a) shows the test image with two Brodatz textures, namely, wood and grass. The segmentation result and error image with white pixels representing misclassifications are shown in Figure 3(b) and Figure 3(c), respectively.  Figure 3(d) shows the percentages of errors at various rates of bits per pixel (bpp). It is noted that the segmentation results even at low-middle bpp rates are still satisfactory. Hence, a small portion of JPEG2000 code stream is sufficient for the segmentation task.

The RDM algorithm has also been extensively evaluated on the Berkeley image database [21]. We adopted the Waveseg algorithm [14] to compute the initial superpixels of a natural color image. In order to avoid decoding a JPEG2000 code stream, the Waveseg algorithm was applied to the estimated wavelet coefficients instead of the decoded wavelet coefficients. More specifically, the estimated wavelet coefficient of *x*_*i*_ using the MQ table-based LPMF is as follows.

**Figure 4:**
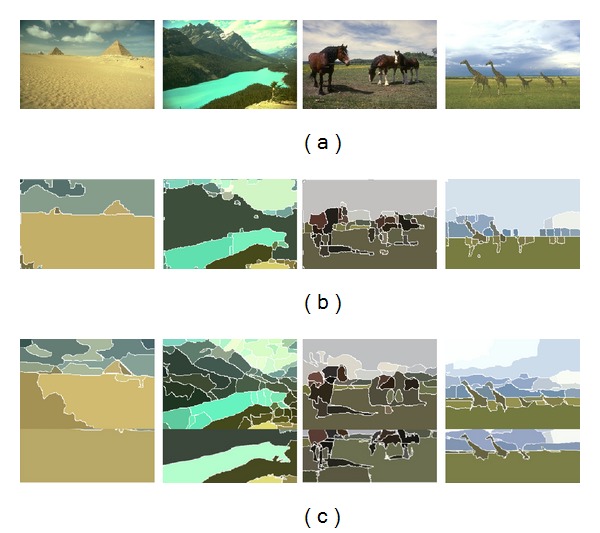
(a) Original images; (b) segmentation using RDM; (c) segmentation using CTM; (d) segmentation using Mean-shift.

(14) E[xi]=∑j=0n−1E[xi,j]·2j=∑j=0n−1Pi,j(xi,j=1)·2j,

where *P*_*i*,*j*_(*x*_*i*,*j*_ = 1) is the probability of 1-bit on the *j*th bit plane, which can be obtained from the MQ table. The resulting superpixels were then merged by RDM with threshold, *T*_*d*_, set to 0.1. We compared the RDM algorithm with two other state-of-the-art algorithms known as Mean-shift [22] and CTM [2]. In Mean-shift, the parameters, *h*_*s*_ and *h*_*r*_, were set to 13 and 19, respectively; in CTM, the threshold *γ* was set to 0.1, as suggested in [2]. The original images shown at the top of Figure 4 are natural images contained in the Berkeley database, namely, Pyramids, Landscape, Horses, and Giraffes. Their respective segmentation results using RDM, CTM, and Mean-shift are shown in the second, third, and fourth rows. Visual inspection shows that RDM and Mean-shift have similar performances for the first three images; the performances of RDM and CTM are similar to detect the giraffes shown in the fourth image.

In addition to visual inspection [23, 24], two commonly used measures, namely, the probabilistic Rand index (PRI) and the boundary displacement error (BDE) [25], were adopted for quantitative comparisons.  Table 1 gives the average PRI performance on the Berkeley database. PRI ranges from 0 to 1, and higher is better. BDE measures the average displacement error of boundaries between segmented images, which is nonnegative, and lower is better. The average BDE performance is given in Table 2. It is noted that RDM outperforms CTM and Mean-shift in terms of the PRI and BDE measures.

The running times on a PC are given in Table 3. It shows that RDM is faster than CTM and Mean-shift due largely to the simple computations of (8) and (13). Moreover, RDM can be applied to a JPEG2000 code stream directly while most algorithms such as Mean-shift and CTM are primarily applied to the original or decoded image and it takes more time to decode a compressed image.

## 5. Conclusions

The MQ table defined in the JPEG2000 standard provides useful information that can be used to compute the local probability mass function (LPMF) of wavelet coefficients. A simple LPMF-based scheme has been proposed to estimate the rate distortion slope (RDS) of a wavelet segment. It is noted that the RDS is increased significantly after merging a pair of wavelet segments with similar characteristics into a single segment. Similar ideas of the above can be used to improve the rate control performance of JPEG2000 [26–28]. In this paper, we propose the rate-distortion-based merging (RDM) algorithm to segment images in the framework of JPEG2000. RDM has been evaluated on images with Brodatz textures and the Berkeley color image database. Experimental results show that the segmentation performance even at low-middle bpp rates is rather promising. For natural images with high-detail contents, RDM is preferable in terms of the average PRI and BDE measures. In addition, the total running time of RDM, which includes the computation of superpixels and the merging process, is faster than Mean-shift and CTM.

As RDM is based on the MQ table, which is available at both encoder and decoder, no overhead transmission is needed to compute the LPMF of wavelet coefficients. RDM can be applied to a JPEG2000 code stream directly; thus, the burden of decompressing computation can be avoided, and memory space that is required to store the decompressed image is no longer necessary from the segmentation point of view.

## Figures and Tables

**Table 1:** Average PRI on the Berkeley database.

RDM	CTM	Mean-shift
0.771	0.762	0.755

**Table 2:** Average BDE on the berkeley database.

RDM	CTM	Mean-shift
8.7	9.4	9.7

**Table 3:** Execution times.

	Pyramids	Landscape	Horses	Giraffes
RDM	8.9 s	8.7 s	10.7 s	6.8 s
Mean-shift	18.3 s	27.5 s	20.7 s	18.9 s
CTM	35.3 s	17.2 s	57.6 s	13.5 s

## References

[1] Xia Y, Feng D, Zhao R (2006). Adaptive segmentation of textured images by using the coupled Markov random field Model. *IEEE Transactions on Image Processing*.

[2] Yang AY, Wright J, Ma Y, Shankar Sastry S (2008). Unsupervised segmentation of natural images via lossy data compression. *Computer Vision and Image Understanding*.

[3] Isa NAM, Salamah SA, Ngah UK (2009). Adaptive fuzzy moving K-means clustering algorithm for image segmentation. *IEEE Transactions on Consumer Electronics*.

[4] Xiang S, Pan C, Nie F, Zhang C (2010). Turbopixel segmentation using eigen-images. *IEEE Transactions on Image Processing*.

[5] Li M, Zhao W (2012). Quantitatively investigating locally weak stationarity of modified multifractional gaussian noise. *Physica A*.

[6] Li M, Zhao W (2010). Variance bound of ACF estimation of one block of fGn with LRD. *Mathematical Problems in Engineering*.

[7] Chen S, Li X (2012). Functional magnetic resonance imaging for imaging neural activity in the human brain: the annual progress. *Computational and Mathematical Methods in Medicine*.

[8] Teng Z, He J, Degnan AJ (2012). Critical mechanical conditions around neovessels in carotid atherosclerotic plaque may promote intraplaque hemorrhage. *Arteriosclerosis, Thrombosis, and Vascular Biology*.

[9] Chen SY, Guan Q (2011). Parametric shape representation by a deformable NURBS model for cardiac functional measurements. *IEEE Transactions on Biomedical Engineering*.

[10] Ilea DE, Whelan PF (2008). CTex—an adaptive unsupervised segmentation algorithm based on color-texture coherence. *IEEE Transactions on Image Processing*.

[11] Mallat S (1999). *A Wavelet Tour of Signal Processing*.

[12] Bashar MK, Ohnishi N, Agusa K (2007). A new texture representation approach based on local feature saliency. *Pattern Recognition and Image Analysis*.

[13] Pun CM, Lee MC (2004). Extraction of shift invariant wavelet features for classification of images with different sizes. *IEEE Transactions on Pattern Analysis and Machine Intelligence*.

[14] Jung CR (2007). Unsupervised multiscale segmentation of color images. *Pattern Recognition Letters*.

[15] Acharya T, Tsai PS (2005). *JPEG2000 Standard for Image Compression: Concepts, Algorithms and VLSI Architectures*.

[16] Cattani C (2010). Harmonic wavelet approximation of random, fractal and high frequency signals. *Telecommunication Systems*.

[17] Chen SY, Wang ZJ (2012). Acceleration strategies in generalized belief propagation. *IEEE Transactions on Industrial Informatics*.

[18] Zargari F, Mosleh A, Ghanbari M (2008). A fast and efficient compressed domain JPEG2000 image retrieval method. *IEEE Transactions on Consumer Electronics*.

[19] Pi MH, Tong CS, Choy SK, Zhang H (2006). A fast and effective model for wavelet subband histograms and its application in texture image retrieval. *IEEE Transactions on Image Processing*.

[20] Hsin HC (2011). Texture segmentation in the joint photographic expert group 2000 domain. *IET Image Processing*.

[21] http://www.eecs.berkeley.edu/~yang/software/lossy_segmentation/.

[22] Comaniciu D, Meer P (2002). Mean shift: a robust approach toward feature space analysis. *IEEE Transactions on Pattern Analysis and Machine Intelligence*.

[23] Hsin HC, Sung T-Y, Shieh Y-S, Cattani C (2012). MQ Coder based image feature and segmentation in the compressed domain. *Mathematical Problems in Engineering*.

[24] Chen S, Zhao M, Wu G, Yao C, Zhang J (2012). Recent advances in morphological cell image analysis. *Computational and Mathematical Methods in Medicine*.

[25] Unnikrishnan R, Pantofaru C, Hebert M (2007). Toward objective evaluation of image segmentation algorithms. *IEEE Transactions on Pattern Analysis and Machine Intelligence*.

[26] Hsin HC, Sung TY (2009). Context-based rate distortion estimation and its application to wavelet image coding. *WSEAS Transactions on Information Science and Applications*.

[27] Hsin H-C, Sung T-Y Image segmentation in the JPEG2000 domain.

[28] Hsin H-C, Sung T-Y, Shieh Y-S, Cattani C (2012). Adaptive binary arithmetic coder-based image feature and segmentation in the compressed domain. *Mathematical Problems in Engineering*.

